# Supplementary material for: Unveiling the Bioactive Potential of the Invasive Jellyfish Phyllorhiza punctata Through Integrative Transcriptomic and Proteomic Analyses
Source: Biomolecules. 2025 Aug 4;15(8):1121. doi: 10.3390/biom15081121 (PMC12383608; doi:10.3390/biom15081121)
Supplement: Supplementary file 1 [file biomolecules-15-01121-s001.zip › Suplementary Material/FileS3/PEPFOLD4/XP_065056240_5270/clusters.html]

 PEP-FOLD clusters


Download archive of all models  here   

|  |  |  |
| --- | --- | --- |
| # | file | sOPEP |
| # cluster 1 - Size: 1 |  |  |
|  | XP\_065056240\_5270-model1 | -23.8187 |
| # cluster 2 - Size: 2 |  |  |
|  | XP\_065056240\_5270-model2 | -21.3356 |
|  | XP\_065056240\_5270-model2.1 | -18.5858 |
| # cluster 3 - Size: 1 |  |  |
|  | XP\_065056240\_5270-model3 | -20.8732 |
| # cluster 4 - Size: 1 |  |  |
|  | XP\_065056240\_5270-model4 | -19.045 |
| # cluster 5 - Size: 1 |  |  |
|  | XP\_065056240\_5270-model5 | -18.9636 |
| # cluster 6 - Size: 1 |  |  |
|  | XP\_065056240\_5270-model6 | -18.6851 |
| # cluster 7 - Size: 2 |  |  |
|  | XP\_065056240\_5270-model7 | -17.9892 |
|  | XP\_065056240\_5270-model7.1 | -15.8504 |
| # cluster 8 - Size: 2 |  |  |
|  | XP\_065056240\_5270-model8 | -17.6952 |
|  | XP\_065056240\_5270-model8.1 | -15.8579 |
| # cluster 9 - Size: 3 |  |  |
|  | XP\_065056240\_5270-model9 | -17.5846 |
|  | XP\_065056240\_5270-model9.1 | -12.7772 |
|  | XP\_065056240\_5270-model9.2 | -12.5096 |
| # cluster 10 - Size: 1 |  |  |
|  | XP\_065056240\_5270-model10 | -17.0208 |
| # cluster 11 - Size: 1 |  |  |
|  | XP\_065056240\_5270-model11 | -16.8538 |
| # cluster 12 - Size: 1 |  |  |
|  | XP\_065056240\_5270-model12 | -16.6323 |
| # cluster 13 - Size: 1 |  |  |
|  | XP\_065056240\_5270-model13 | -16.3546 |
| # cluster 14 - Size: 1 |  |  |
|  | XP\_065056240\_5270-model14 | -16.1106 |
| # cluster 15 - Size: 2 |  |  |
|  | XP\_065056240\_5270-model15 | -16.0425 |
|  | XP\_065056240\_5270-model15.1 | -11.9353 |
| # cluster 16 - Size: 1 |  |  |
|  | XP\_065056240\_5270-model16 | -15.7615 |
| # cluster 17 - Size: 3 |  |  |
|  | XP\_065056240\_5270-model17 | -15.7532 |
|  | XP\_065056240\_5270-model17.1 | -14.1367 |
|  | XP\_065056240\_5270-model17.2 | -12.2933 |
| # cluster 18 - Size: 3 |  |  |
|  | XP\_065056240\_5270-model18 | -15.6809 |
|  | XP\_065056240\_5270-model18.1 | -13.6132 |
|  | XP\_065056240\_5270-model18.2 | -12.3695 |
| # cluster 19 - Size: 1 |  |  |
|  | XP\_065056240\_5270-model19 | -15.184 |
| # cluster 20 - Size: 1 |  |  |
|  | XP\_065056240\_5270-model20 | -15.0825 |
| # cluster 21 - Size: 2 |  |  |
|  | XP\_065056240\_5270-model21 | -14.9909 |
|  | XP\_065056240\_5270-model21.1 | -12.6304 |
| # cluster 22 - Size: 1 |  |  |
|  | XP\_065056240\_5270-model22 | -14.8089 |
| # cluster 23 - Size: 2 |  |  |
|  | XP\_065056240\_5270-model23 | -14.7613 |
|  | XP\_065056240\_5270-model23.1 | -14.3397 |
| # cluster 24 - Size: 1 |  |  |
|  | XP\_065056240\_5270-model24 | -14.6436 |
| # cluster 25 - Size: 1 |  |  |
|  | XP\_065056240\_5270-model25 | -14.5113 |
| # cluster 26 - Size: 1 |  |  |
|  | XP\_065056240\_5270-model26 | -14.5076 |
| # cluster 27 - Size: 1 |  |  |
|  | XP\_065056240\_5270-model27 | -14.4763 |
| # cluster 28 - Size: 1 |  |  |
|  | XP\_065056240\_5270-model28 | -14.3651 |
| # cluster 29 - Size: 1 |  |  |
|  | XP\_065056240\_5270-model29 | -14.2705 |
| # cluster 30 - Size: 2 |  |  |
|  | XP\_065056240\_5270-model30 | -14.0814 |
|  | XP\_065056240\_5270-model30.1 | -11.9538 |
| # cluster 31 - Size: 1 |  |  |
|  | XP\_065056240\_5270-model31 | -14.0623 |
| # cluster 32 - Size: 1 |  |  |
|  | XP\_065056240\_5270-model32 | -14.0329 |
| # cluster 33 - Size: 1 |  |  |
|  | XP\_065056240\_5270-model33 | -14.0006 |
| # cluster 34 - Size: 1 |  |  |
|  | XP\_065056240\_5270-model34 | -13.9894 |
| # cluster 35 - Size: 1 |  |  |
|  | XP\_065056240\_5270-model35 | -13.914 |
| # cluster 36 - Size: 1 |  |  |
|  | XP\_065056240\_5270-model36 | -13.909 |
| # cluster 37 - Size: 1 |  |  |
|  | XP\_065056240\_5270-model37 | -13.8857 |
| # cluster 38 - Size: 1 |  |  |
|  | XP\_065056240\_5270-model38 | -13.7981 |
| # cluster 39 - Size: 1 |  |  |
|  | XP\_065056240\_5270-model39 | -13.7908 |
| # cluster 40 - Size: 1 |  |  |
|  | XP\_065056240\_5270-model40 | -13.7795 |
| # cluster 41 - Size: 1 |  |  |
|  | XP\_065056240\_5270-model41 | -13.6829 |
| # cluster 42 - Size: 1 |  |  |
|  | XP\_065056240\_5270-model42 | -13.6536 |
| # cluster 43 - Size: 1 |  |  |
|  | XP\_065056240\_5270-model43 | -13.5491 |
| # cluster 44 - Size: 1 |  |  |
|  | XP\_065056240\_5270-model44 | -13.5273 |
| # cluster 45 - Size: 1 |  |  |
|  | XP\_065056240\_5270-model45 | -13.5272 |
| # cluster 46 - Size: 1 |  |  |
|  | XP\_065056240\_5270-model46 | -13.4568 |
| # cluster 47 - Size: 1 |  |  |
|  | XP\_065056240\_5270-model47 | -13.4113 |
| # cluster 48 - Size: 1 |  |  |
|  | XP\_065056240\_5270-model48 | -13.368 |
| # cluster 49 - Size: 1 |  |  |
|  | XP\_065056240\_5270-model49 | -13.3332 |
| # cluster 50 - Size: 2 |  |  |
|  | XP\_065056240\_5270-model50 | -13.2978 |
|  | XP\_065056240\_5270-model50.1 | -12.4537 |
| # cluster 51 - Size: 2 |  |  |
|  | XP\_065056240\_5270-model51 | -13.2372 |
|  | XP\_065056240\_5270-model51.1 | -13.0211 |
| # cluster 52 - Size: 1 |  |  |
|  | XP\_065056240\_5270-model52 | -13.2183 |
| # cluster 53 - Size: 1 |  |  |
|  | XP\_065056240\_5270-model53 | -13.1957 |
| # cluster 54 - Size: 1 |  |  |
|  | XP\_065056240\_5270-model54 | -13.1035 |
| # cluster 55 - Size: 1 |  |  |
|  | XP\_065056240\_5270-model55 | -13.0935 |
| # cluster 56 - Size: 1 |  |  |
|  | XP\_065056240\_5270-model56 | -13.0883 |
| # cluster 57 - Size: 1 |  |  |
|  | XP\_065056240\_5270-model57 | -13.057 |
| # cluster 58 - Size: 2 |  |  |
|  | XP\_065056240\_5270-model58 | -12.923 |
|  | XP\_065056240\_5270-model58.1 | -12.0969 |
| # cluster 59 - Size: 1 |  |  |
|  | XP\_065056240\_5270-model59 | -12.917 |
| # cluster 60 - Size: 1 |  |  |
|  | XP\_065056240\_5270-model60 | -12.8613 |
| # cluster 61 - Size: 1 |  |  |
|  | XP\_065056240\_5270-model61 | -12.8592 |
| # cluster 62 - Size: 1 |  |  |
|  | XP\_065056240\_5270-model62 | -12.8351 |
| # cluster 63 - Size: 1 |  |  |
|  | XP\_065056240\_5270-model63 | -12.7431 |
| # cluster 64 - Size: 1 |  |  |
|  | XP\_065056240\_5270-model64 | -12.6491 |
| # cluster 65 - Size: 1 |  |  |
|  | XP\_065056240\_5270-model65 | -12.625 |
| # cluster 66 - Size: 1 |  |  |
|  | XP\_065056240\_5270-model66 | -12.6232 |
| # cluster 67 - Size: 1 |  |  |
|  | XP\_065056240\_5270-model67 | -12.6136 |
| # cluster 68 - Size: 1 |  |  |
|  | XP\_065056240\_5270-model68 | -12.5781 |
| # cluster 69 - Size: 1 |  |  |
|  | XP\_065056240\_5270-model69 | -12.5466 |
| # cluster 70 - Size: 1 |  |  |
|  | XP\_065056240\_5270-model70 | -12.5285 |
| # cluster 71 - Size: 1 |  |  |
|  | XP\_065056240\_5270-model71 | -12.4936 |
| # cluster 72 - Size: 1 |  |  |
|  | XP\_065056240\_5270-model72 | -12.4491 |
| # cluster 73 - Size: 1 |  |  |
|  | XP\_065056240\_5270-model73 | -12.3813 |
| # cluster 74 - Size: 1 |  |  |
|  | XP\_065056240\_5270-model74 | -12.2989 |
| # cluster 75 - Size: 1 |  |  |
|  | XP\_065056240\_5270-model75 | -12.2399 |
| # cluster 76 - Size: 1 |  |  |
|  | XP\_065056240\_5270-model76 | -12.225 |
| # cluster 77 - Size: 1 |  |  |
|  | XP\_065056240\_5270-model77 | -11.9914 |
| # cluster 78 - Size: 1 |  |  |
|  | XP\_065056240\_5270-model78 | -11.8209 |
| # cluster 79 - Size: 2 |  |  |
|  | XP\_065056240\_5270-model79 | -11.7975 |
|  | XP\_065056240\_5270-model79.1 | -11.6377 |
| # cluster 80 - Size: 1 |  |  |
|  | XP\_065056240\_5270-model80 | -11.6367 |
| # cluster 81 - Size: 1 |  |  |
|  | XP\_065056240\_5270-model81 | -11.5673 |
| # cluster 82 - Size: 1 |  |  |
|  | XP\_065056240\_5270-model82 | -11.2559 |
| # cluster 83 - Size: 1 |  |  |
|  | XP\_065056240\_5270-model83 | -10.4363 |
|  |  |  |
